# Linkage Disequilibrium between Two High-Frequency Deletion Polymorphisms: Implications for Association Studies Involving the *glutathione-S transferase* (*GST*) Genes

**DOI:** 10.1371/journal.pgen.1000472

**Published:** 2009-05-08

**Authors:** Yongzhong Zhao, Michael Marotta, Evan E. Eichler, Charis Eng, Hisashi Tanaka

**Affiliations:** 1 Department of Molecular Genetics, Cleveland Clinic, Cleveland, Ohio, United States of America; 2 Lerner Research Institute, Cleveland Clinic, Cleveland, Ohio, United States of America; 3 Department of Genome Sciences, University of Washington, Seattle, Washington, United States of America; 4 Howard Hughes Medical Institute, University of Washington, Seattle, Washington, United States of America; 5 Genomic Medicine Institute, Cleveland Clinic, Cleveland, Ohio, United States of America; 6 Taussig Cancer Institute, Cleveland Clinic, Cleveland, Ohio, United States of America; 7 Department of Genetics, Case Western Reserve University School of Medicine, Cleveland, Ohio, United States of America; 8 CASE Comprehensive Cancer Center, Case Western Reserve University School of Medicine, Cleveland, Ohio, United States of America; Wellcome Trust Sanger Institute, United Kingdom

## Abstract

Copy number variations (CNVs) represent a large source of genetic variation in humans and have been increasingly studied for disease association. A deletion polymorphism of the gene encoding the cytosolic detoxification enzyme glutathione S-transferase theta 1 (*GSTT1*) has been extensively studied for cancer susceptibility (919 studies, from HuGE navigator, http://www.hugenavigator.net/). However, clear conclusions have not been reached. Since the *GSTT1* gene is located within a genomic region of segmental duplications (SD), there may be a confounding effect from another, yet-uncharacterized CNV at the same locus. Here we describe a previously uncharacterized 38-kilo-base (kb) long deletion polymorphism of *GSTT2B* located within a 61-kb DNA inverted repeat. *GSTT2B* is a duplicated copy of *GSTT2*, the only paralogue of *GSTT1* in humans. A newly developed PCR assay revealed that a microhomology-mediated breakpoint appears to be shared among individuals at high frequency. The *GSTT2B* deletion polymorphism was in strong linkage disequilibrium (LD) (D′ = 0.841) with the neighboring *GSTT1* deletion polymorphism in the Caucasian population. Alleles harboring a single deletion were significantly overrepresented (p = 2.22×10^−16^), suggesting a selection against alleles with both deletions. The deletion alleles are almost certainly the derived ones, because the *GSTT2B-GSTT2-GSTT1* genes were strictly retained in chimpanzees. Extremely low *GSTT2* mRNA expression was associated with the *GSTT2B* deletion, suggesting an influence of the deletion on the flanking region and loss of *GSTT2* function. Genome-wide LD analysis between deletion polymorphisms further points to the uniqueness of two deletions, because strong LD between deletion polymorphisms might be very rare in humans. These results show a complex genomic organization and unexpected biological functions of CNVs within segmental duplications and emphasize the importance of detailed structural characterization for disease association studies.

## Author Summary

Common diseases such as cancer are caused by interactions between multiple genetic and environmental factors. Glutathione S-transferases (GST) are key enzymes in eliminating carcinogens and harmful macromolecules from cells. Based on the assumption that individuals who do not have a particular type of *GST* genes are susceptible to cancers, a number of studies have been conducted to find a link between *GST* genotypes and cancer. However such associations remain inconclusive to date. Because *GST* genes are clustered in repetitive, complex regions in the genome, other previously uncharacterized variations/polymorphisms may have had an impact on the data. We describe here such a genotype, a 37-kb deletion of *GSTT2B* gene that is found very frequently among humans. The neighboring *GSTT2* gene expression is greatly impaired by the *GSTT2B* deletion, conferring a potentially null allele at *GSTT2*. The *GSTT2B* deletion is non-randomly associated with another high frequency deletion of the *GSTT1* gene. Therefore, a detailed characterization of this complex region of the genome revealed unexpected genetic and biological interactions of large deletion polymorphisms; this is essential to consider in future disease association studies.

## Introduction

Copy number variation (CNV) is a significant source of genetic variation in the genome of humans [1]–[11]. A large number of CNVs has been identified, and span more than 10% of the human genome in total [12], although the estimate is dependent on the frequency of the event under consideration. The biomedical relevance of CNVs is expected to be significant, because many CNVs cover large genomic regions and include exons and regulatory elements that are important for proper cellular function. However, these CNVs are primarily identified by indirect, array-based methods with limited resolution; defining fine scale structure, especially for large CNVs, is just beginning at the sequence level [9],[13],[14]. Without such information, it is difficult to determine each CNV's history, population structure, and influence on the function of one or more genes within the CNV and surrounding genomic regions.

CNVs are significantly enriched in the regions of segmental duplications (SD) [6]–[8],[10],[12]. SDs are highly identical DNA segments that map to two or more loci within the genome [15],[16]. Since regions of SDs have strong positive correlations with genes [15],[17], CNVs that overlap with SDs are particularly gene-rich. Therefore, defining the extent and breakpoint in each CNV in regions of SD is particularly important in order to identify CNVs that may have clinical relevance. In fact, CNVs are highly enriched in gene classes such as defense and immune response [1],[18], suggesting a link between CNVs in SDs and human health. However, determining the detailed structures of CNVs in SDs is not an easy task. First, given the fact that DNA sequences in SDs vary substantially among individuals, any technology based on the reference genome sequence may not be sufficient to accurately map all CNVs. Second, single nucleotide polymorphisms (SNPs), the most widely used markers to tag genomic locations, are not always reliable within SDs [19],[20]. Although SNP-based methods have identified a large number of deletion polymorphisms successfully [1],[5], this approach may not be as efficient in SDs as within unique segments of the genome. Therefore, more direct approaches, such as clone-based sequencing for mapping breakpoints, and subsequent molecular assays for genotyping, are necessary to accurately interrogate CNVs in regions of SDs [21].

The importance of CNVs in human diseases has become increasingly apparent [22],[23]. It has long been known that DNA rearrangements of large genomic regions play a major role in the pathogenesis of rare genetic diseases (genomic disorders) [24]–[26], and more recently, more common complex diseases such as non-syndromic mental retardation, autism and schizophrenia [27]–[30]. Common deletion polymorphisms of a class of genes in cellular detoxification, glutathion S-transferases (GSTs), have also been known for more than a decade [31],[32]. *GST* is a supergene family. Each sub-family member is located in a distinct genomic region and consists of as many as five paralogues [33]. *GST* gene products catalyze the conjugation of reduced glutathione to electrophilic centers for a wide variety of substrates [34]. The increased solubility of the conjugated products renders them more readily eliminated by the cell. Substrates include both xenobiotics and endogenous compounds that are harmful to cellular macromolecules. Based on the hypothesis that lack of GST may cause reduced levels of cellular detoxification, and thus predispose individuals to common diseases such as cancer, previously defined null alleles (deletion polymorphisms) have been subject to extensive disease-association studies (1230 published studies, information obtained from HuGE Navigator). However, to date, the reports contain conflicting results [35]–[38]. One possible explanation for the conflict could be that due to extensive segmental duplications in the genomic loci of GST family members, there are other, yet-uncharacterized null alleles that may impact the results.

**Figure 1:**
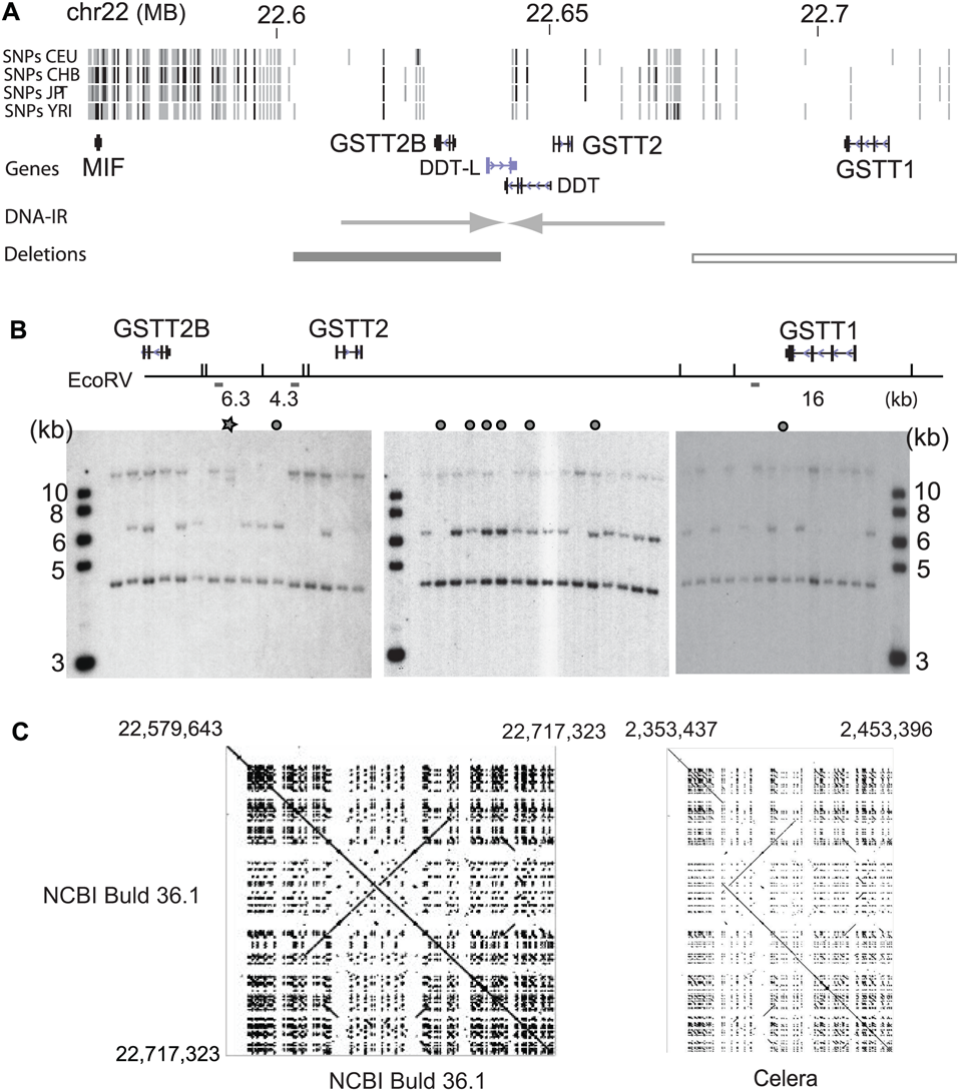
Deletion polymorphisms of the *GSTT2B* and *GSTT1* genes. A. 150-kb genomic locus harboring the *GSTT2B*, *GSTT2* and *GSTT1* genes. Information on HapMap SNPs and known genes were obtained from UCSC genome browser. The location of a 61 kb DNA inverted repeats (indicated by converging arrow heads) was based on the information from IRDB database. The location of *GSTT2B* deletion polymorphism is based on the sequence information obtained in this study. The location of GSTT1 deletion polymorphism is based on the sequence information from Sprenger *et al.* Black rectangle represents a location of the *GSTT2B* deletion polymorphism. Open rectangle indicates the *GSTT1* deletion polymorphism. B. Southern blotting analysis with a probe for three EcoRV fragments. Restriction map with the locations of the *GSTT2B*, *GSTT2* and *GSTT1* gene is shown. The probe (a small rectangle) hybridized to three fragments: left repeat of the DNA-IR (6.3 kb), right repeat of the DNA IR (4.3 kb) and the fragment near *GSTT1* (16 kb). Results of Southern hybridization from 44 individuals (38 Caucasians and 6 from other populations, marked by gray circles) are shown. Note that many individuals do not have the 6.3 kb fragment. C. Genome assembly comparison by Pipmaker. (*Left*), self alignment of the 137 kb genomic sequence from the NCBI Build 36.1. Coordinates are shown on the Y axis. A diagonal indicates that the same DNA sequences plotted on the x and y-axis. Note that a large DNA inverted repeat (a crossing line to the diagonal) exists within this genomic region. (*Right*), Assembly comparison between the Build 36.1 (y axis) and Celera assembly (x axis). The right half of DNA inverted repeat is missing in the Celera assembly, which is shown as a discontinuous diagonal and a duplicated sequence present only in the Build 36.1.

In this study, using DNA samples from blood, lymphoblastoid cell lines, HapMap populations, and chimpanzees; and RNA from primary fibroblasts and cancer cell lines, we conducted a systematic genetic, gene expression and evolutionary analysis for a previously uncharacterized large deletion polymorphism located at chromosome 22q13, a genomic region with a 61 kilo-base (kb) inverted repeat. Each repeat harbors a theta class of *GST* gene, *GSTT2B* on the centromeric side of the repeat and *GSTT2* on the telomeric side (Figure 1A). A 37-kb deletion encompassed s the entire centromeric side and the *GSTT2B* gene. We show here that the deletion allele is very common in all three HapMap populations. In particular, a high frequency deletion allele (66%) in the CEU population is in linkage disequilibrium (LD) with the neighboring *GSTT1* deletion polymorphism. Such a strong LD between deletion polymorphisms is indeed very rare within the currently known deletion polymorphisms. The deletion has a strong influence on the remaining *GSTT2*, as we found that *GSTT2* expression is severely reduced in cells with homozygous deletion of *GSTT2B*. SNP analysis within the deletion region, however, failed to yield null genotypes, possibly because almost all these SNPs are located within a recently duplicated region.

## Results

### Frequent deletion polymorphism associated with a large DNA inverted repeat

To identify structural variation in the regions of large DNA inverted repeats (DNA-IR), we first obtained information of DNA-IRs represented in the human genome sequence (Build 35) from the Inverted Repeat Database (IRDB) [39]. Because of secondary structures, perfect DNA palindromes, with small non-palindromic spacers between arms (repeats), are predisposed to DNA rearrangements in both simple organisms and mammals [40],[41]. Therefore, we hypothesized that large DNA-IRs with high-sequence identity between repeats and small non-palindromic spacers may often be subject to chromosome breakage and DNA rearrangement, and, as a result, likely to be enriched for structural variations. Among large DNA-IRs in the human genome, one on the chromosome 22q11.23 has a large repeat unit size (29.6-kb) with 97.9% sequence identity between repeats, and a 2.1-kb spacer (Figure 1A). This DNA-IR has previously been shown to be located in the region of discordance by fosmid end-mapping and copy number variation analyses [6],[9]. Other features are also notable in this region, such as a high frequency deletion polymorphism (*GSTT1*, Figure 1A open rectangle), and a low density of the HapMap SNPs. The gene duplicated in the DNA-IR is *GSTT2*, a theta class glutathione transferase. We use the gene name *GSTT2B* for the *GSTT2* located on the centromeric (left) repeat according to the annotation in the UCSC genome browser.

Molecular characterization of DNA-IRs is a challenge, because DNA-IRs with small spacers are known to be resistant to PCR amplification and cloning in E.coli. Southern analysis and restriction fragment length polymorphism has been successfully used to determine DNA structure within DNA-IRs [42]. To identify a structural variation associated with the DNA-IR, we designed a probe that was hybridized to the DNA near the non-palindromic spacer. DNA rearrangements are known to occur most frequently at the spacer and surrounding regions [43]. We also took advantage of the segmentally duplicated sequences in this locus. We designed a probe with high sequence homology to the three regions (Figure 1B). By using restriction enzyme EcoRV, we could determine genotypes for both *GSTT1* and *GSTT2* simultaneously. EcoRV-digested genomic DNA samples of lymphoblastoid cell lines established from 38 Caucasian individuals were used to determine the lengths of three restriction fragments, including a 4.6-kb fragment on the telomeric (right) repeat of the DNA-IR, a 6.3-kb fragment on the centromeric (left) repeat, and a 16 kb fragment near the *GSTT1* gene. As is shown in Figure 1B, the 6.3 kb fragment was very frequently missing in these samples. Nineteen samples did not have the 6.3-kb fragment, suggesting a homozygous deletion of the right repeat of DNA-IR. The deletion was further confirmed by using genomic DNA digested with both SfiI and NdeI (Figure S1). In addition to the potential homozygous deletion, there were samples that showed reduced intensity of the 6.3 kb fragment relative to the 4.6 kb one. These individuals could be heterozygous for the deletion. Furthermore, the 16-kb fragments were not seen in 9 individuals, suggesting a homozygous deletion of the *GSTT1* gene. Finally, a unique 10 kb fragment is seen in one individual (Figure 1B, star).

**Figure 2:**
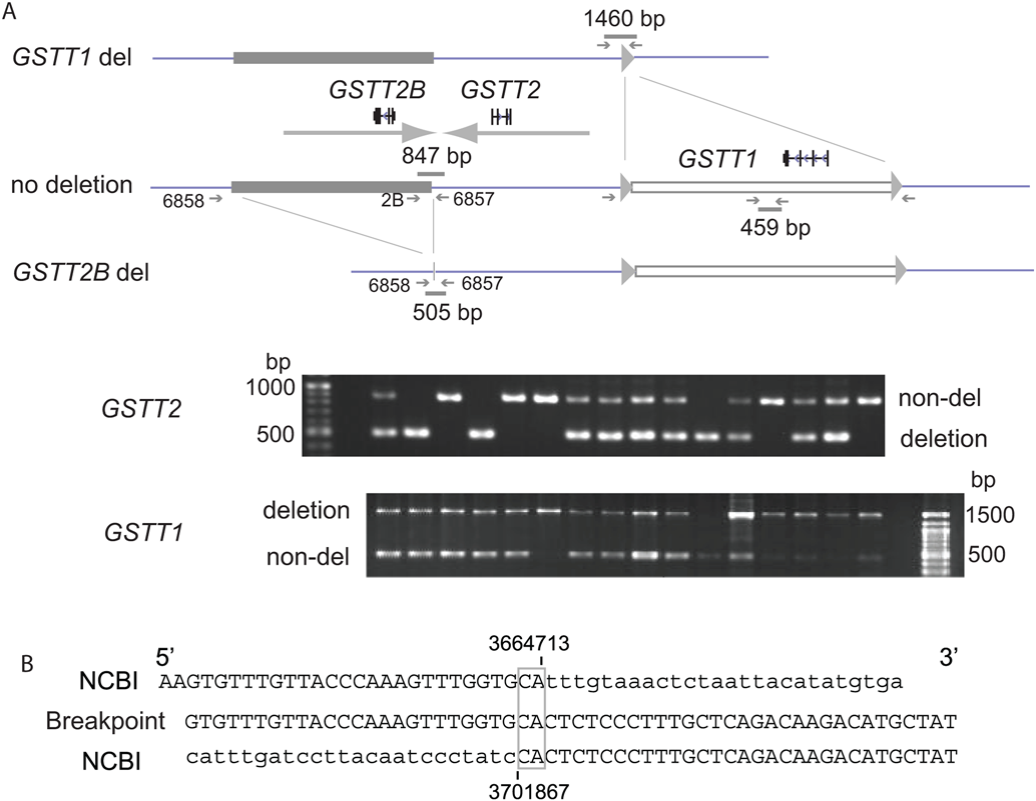
PCR assay for the *GSTT2B* and *GSTT1* deletion. A. Solid lines represent genomic sequences, and rectangles represent deleted sequences. The locations of genes (*GSTT2B*, *GSTT2* and *GSTT1*) and a DNA-IR are shown. Expected PCR products are drawn as small gray bars. A PCR assay for the *GSTT2B* deletion amplifies a 847 bp fragments for the non-deletion allele (middle), while a 505 bp fragment is amplified for the deletion allele (bottom). A PCR assay for the *GSTT1* deletion, developed by Strenger et al [45]., amplifies a 466 bp for non-deletion allele (middle), while a 1460 bp product is expected for deletion allele (top). A small gray triangle indicates the 408-bp repeat flanking *GSTT1* deletion. Results from the two PCR assays for 16 Caucasians are shown. B. A microhomology-mediated breakpoint. DNA sequence of the breakpoint (in the middle) is aligned with the Build 36 (top and bottom, with each coordinates). Note that there is a two-bp microhomology at the breakpoint (in the open rectangle). Capital letter represents sequences that are present in both assemblies.

Southern analysis above clearly illustrated a frequent deletion and complex pattern of structural variation within and near the 61-kb DNA-IR. To determine the extent and breakpoint of deletion, genome assembly comparison was performed between the NCBI Build 36 and Celera assembly (Figure 1C). To identify differences at sequence-level resolution, we directly compared DNA sequences by PipMaker [44]. The DNA sequences used for this comparison cover the genomic region between *MIF* and *GSTT1*. Self-comparison of the NCBI assembly showed a large DNA-IR that was illustrated by a large cross-line (left) to the main diagonal. In contrast, there was sequence discordance at the region of the DNA-IR between two assemblies (right). Thirty-seven kb of genomic sequences, including an entire left repeat of the DNA-IR was missing in the Celera assembly. In fact, the DNA-IR was not seen in the dot plot created by the self-comparison of Celera assembly (data not shown). In order to determine whether the frequent deletion observed by Southern analysis was represented in the Celera assembly, a PCR primer set was designed to amplify a putative breakpoint (Figure 2A). This primer set amplified the 505-bp fragment from the *GSTT2B* deletion allele (del), but could not amplify a product of 39-kb (deleted region plus franking sequence) from the non-deleted allele. A PCR product of expected size was seen from the individuals that show a missing or reduced intensity of a 6.3-kb fragment. DNA sequencing of the PCR products form 5 individuals showed that an identical breakpoint was shared among individuals. The breakpoint resided within a unique (non-repetitive) sequence and was mediated by 2-bp microhomology (Figure 2B). From these results, we predicted that a *GSTT2B*-deleted allele exists at high frequency in our Caucasian samples. This allele may also be a common one in human populations, because (1) this allele is represented in the Celera assembly and (2) the breakpoint was identified by recent paired end-pair mappings with a small number of samples [13],[14].

### Common *GSTT2B* deletion polymorphism is in linkage disequilibrium with neighboring *GSTT1* deletion

A 37-kb *GSTT2B* deletion polymorphism was located very close to another 54-kb deletion polymorphism of *GSTT1*. Thus, two large, high-frequency deletion polymorphisms exist within a genomic region of 124 kb. CNVs are very common in the human genome. However, neighboring, large, high frequency deletions could be relatively rare occurrences. In order to identify whether the deletion genotype is found at a high frequency in a large sample population, we developed a PCR-based assay (Figure 2A). Three primer sets were designed to simultaneously PCR-amplify both the non-deleted (847-bp) and deleted allele (505 bp) of *GSTT2B*. Similarly, previously developed PCR assay was used to detect the *GSTT1* deletion [45]. These PCR-based assays were first applied to the genomic DNA from blood samples of the same Caucasian population that we used for screening by Southern analysis. To determine the robustness of our PCR-based assay to detect the *GSTT2B* deletion, we genotyped these samples using both Southern analysis and our PCR-based assay in a blinded manner. The results obtained by both methods were then unblended and revealed almost complete concordance (37/39 individuals). The two cases (2 individuals, 5%) of discordance could be due to either the less accurate calling based on the relative intensity between the 4.3- and 6.3-kb fragments by Southern analysis, or the existence of CNV with distinct breakpoints (Figure 1B, star). The frequency of the *GSTT2B* deletion was very high in the population analyzed; deletion allele frequency (0.54) was higher than that of non-deletion allele (0.46) (Table 1). The allele frequency of the *GSTT1* deletion was 0.36, which was comparable to the frequency in the CEU population (0.39) of the HapMap samples [5].

**Table 1:** GSTT2B and GSTT1 deletion polymorphisms in 38 Caucasian individuals.

Genotype				
Gene	Del/Del	non-del/non-del	Del/Non-del	HWE(p-value)
*GSTT2B (n = 38)*	13	11	14	0.1121
*GSTT1 (n = 38)*	6	17	15	0.4780

Population	Haplotype	Freq	S.E	D	D′	Corr	Chisq	P-value
CEU-38-sample				0.1479	0.7719	0.6201	29.2277	6.44E-08
	GSTT2B_DEL-GSTT1	0.4941	0.0093					
	GSTT2B_DEL-GSTT1_DEL	0.0454	0.0093					
	GSTT2B-GSTT1	0.1638	0.0093					
	GSTT2B-GSTT1_DEL	0.2967	0.0093					

Freq, allele frequency; S.E., standard error; D, raw difference in frequencey between observed number and expected number; D′, scaled D spanning the range [−1,1]; Corr, Correlation Coefficient; chisq, Chi-square statistics for linkage equilibrium; p-value, Chi-square p-value for marker independence.

From the Southern analysis, we noticed a potential linkage between the two deletion polymorphisms. Individuals who did not have the 6.3-kb fragment tended to have the 16-kb fragment, and individuals who did not have the 16 kb fragment tended to have the 6.3-kb fragment. This suggests a non-random assortment (Linkage Disequilibrium, LD) between the two deletion polymorphisms. In order to assess LD between the deletions, we reconstructed deletion-based haplotypes using PHASE [46] (Table 1). Each deletion genotype was determined based on the results from the PCR-based assay. Haplotype frequencies at the locus were found to be significantly deviated from the expected values: single-gene deletions were overrepresented whereas alleles with both gene deletions were exceedingly rare (p = 5.17×10^−7^). The frequency of the *GSTT2* deletion/*GSTT1* non-deletion haplotype was 0.49 (expected 0.34, if random) while the frequency of the *GSTT2* non-deletion/*GSTT1* deletion was 0.29 (0.165, if random). The frequency of the haplotype with both deletions was very low, 0.048 (0.19, if random). Thus, high frequency, neighboring deletion polymorphisms were non-randomly associated in Caucasian populations (*D′* = 0.7719).

### Extremely low *GSTT2* mRNA expression with the *GSTT2B* deletion

**Figure 3:**
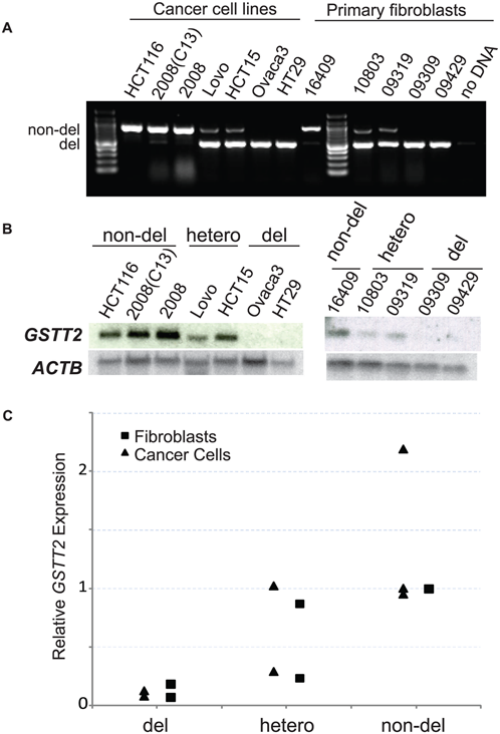
Very low level of *GSTT2* mRNA expression with the *GSTT2B* deletion. A. GSTT2B genotype analysis for the cancer cell lines and primary fibroblasts used for gene expression analysis. B. Northern blot analysis of the *GSTT2* gene expression. Results from cancer cell lines (left) and primary fibroblasts (right) are shown. Northern blot with the human β-actin gene probe are shown as a control. Genotypes are indicated on the top. Note that gene expression levels for cells with homozygous deletion are very low. C. Real time PCR analysis for *GSTT2* expression. Gene expression was normalized to HCT116 (for cancer cell lines) and AG16409 (for primary fibroblasts). Relative expression level of each cancer cell line (closed circle) and primary fibroblast (black triangle) are shown.

The *GSTT2B* deletion was not expected to have an effect on *GSTT2* expression, because the *GSTT2* gene and its promoter regions were intact in the *GSTT2B*-deleted allele. Gene expression levels can be proportionate to the gene dosage in the case of exonic deletions [5], in which case, we should expect a half level of *GSTT2* expression. Alternatively, a large genomic deletion may influence the level of *GSTT2* expression. To determine the potential effect of *GSTT2B* deletion on *GSTT2* expression, we measured the *GSTT2* mRNA expression level for each genotype. *GSTT2* was not expressed at an appreciable level in the lymphoblastoid cell lines and was undetectable by Northern analysis. Therefore, we first examined 7 cancer cell lines that included three cell lines homozygous for the non-deletion allele (HCT116, 2008-C13, and 2008), two heterozygous (Lovo and HCT15) and two cell lines homozygous for the deletion allele (Ovaca3 and HT29) (Figure 3A). *GSTT2* expression was readily detectable in cell lines with the *GSTT2B* non-deletion allele. In contrast, in cell lines with homozygous deletions of *GSTT2B*, *GSTT2* expression was undetectable (Figure 3B).

Cancer cell lines are very often aneuploid, which may contribute to the observed pattern of gene expression. We further determined *GSTT2* gene expression using 5 primary fibroblasts. Consistent with the results from cancer cell lines, *GSTT2* expression was strong in a fibroblast homozygous for the non-deletion allele, was weaker but detectable when heterozygous, and was undetectable in cell lines homozygous for the *GSTT2B* deletion. Finally, quantitative RT-PCR analysis (Figure 3C) showed relative gene expression levels that are very similar to the pattern observed for null and non-null genotype; cells homozygous for the *GSTT2B* deletion showed more than 80% reduction of *GSTT2* expression in cell lines homozygous for the non-deletion alleles. Therefore, a large deletion including *GSTT2B* influences the expression of a flanking gene and correlates with the very low level of *GSTT2* mRNA expression.

### *GSTT2B* and *GSTT1* deletion polymorphism as human specific CNVs

**Figure 4:**
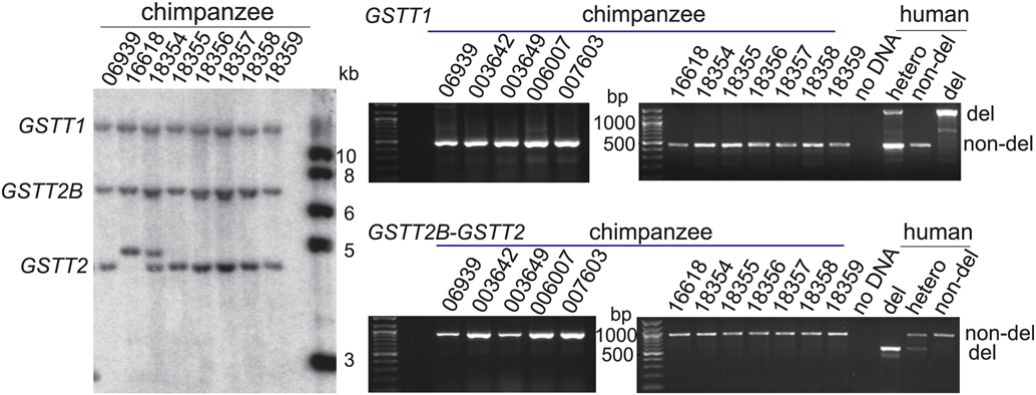
Characterization of *GSTT2B*-*GSTT2*-*GSTT1* locus in chimpanzee. (*Left*), Southern analysis for eight chimpanzee cell lines. EcoRV digested genomic DNA was hybridized with the same probe used for human samples in Figure 1B. Note that there is the same pattern of restriction fragments in chimpanzees as in humans, with the fragments of 4.3 kb, 6.3 kb and 16 kb that correspond to the fragments representing *GSTT2*, *GSTT2B* and *GSTT1* in human respectively (Figure 1B). (*Right*), PCR genotyping for 12 chimpanzee samples. Non-deletion alleles for both *GSTT2* and *GSTT1* were exclusively observed. PCR products from human samples were shown as examples of the deletion, heterozygous and non-deletion genotypes.

We predicted two possible ancestral allelic states for the *GSTT2B*-*GSTT2* region: 1) a single *GSTT2* gene that is duplicated during the evolution of humans, or 2) an inverted duplication that was in part deleted in the human lineage. In principle, the ancestral allele can be inferred by analysis of the chimpanzee genome sequence assembly (panTro2). However, we were unable to determine the ancestral state due to the over-abundance of gaps surrounding the chimpanzee *GSTT2* assembly. Instead, we applied molecular analyses that determined genotypes on human samples (Figure 4). Three restriction fragments representing *GSTT2B*, *GSTT2* and *GSTT1* in humans were all conserved in 12 chimpanzee samples, with an exception of a polymorphism seen in the 4.6-kb fragment. The results from PCR-based assays were also consistent with the non-deletion state of both *GSTT1* and *GSTT2B* in the chimpanzee. Therefore, the ancestral state is most likely a duplicated *GSTT2*, where both of the deletion alleles are derived within the human lineage.

### SNP genotypes within a DNA inverted repeat

**Figure 5:**
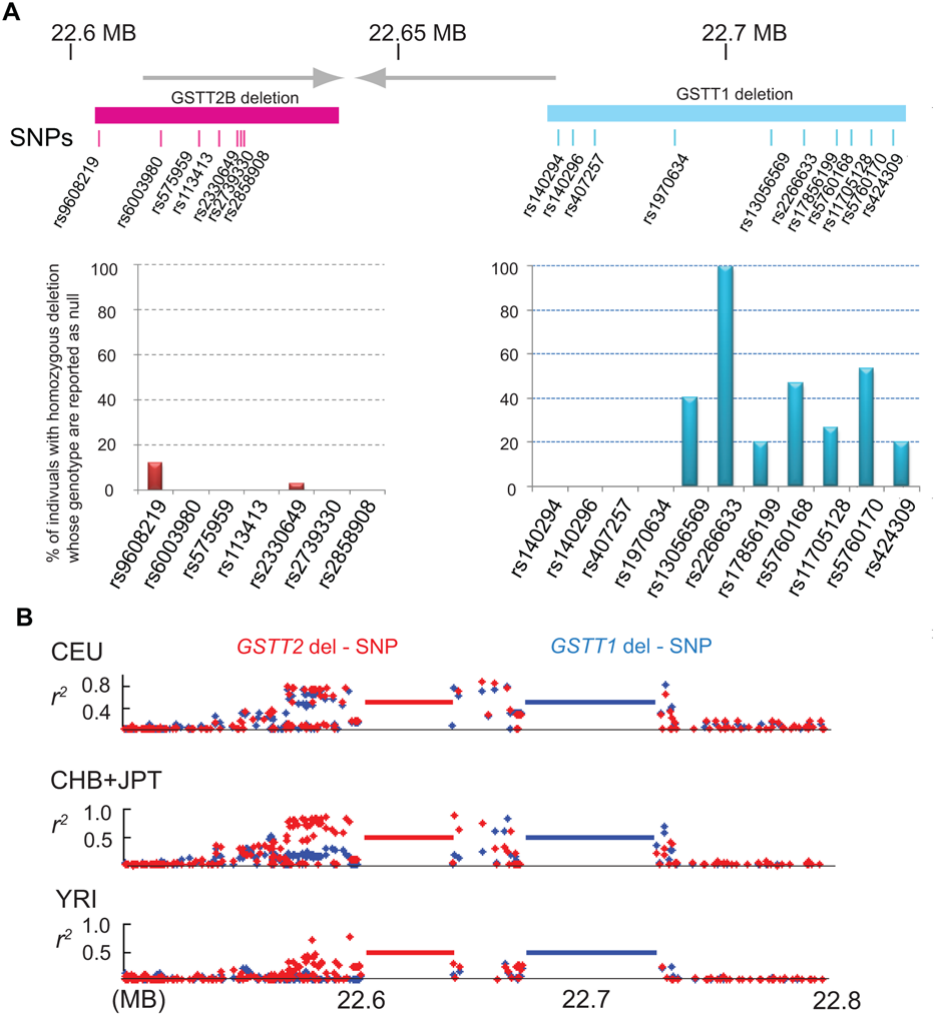
SNP genotypes within a complex locus. A. Failed SNP genotyping in a recently duplicated segment. The locations of SNPs within the *GSTT2B* deletion (red) and *GSTT1* deletion (light blue) are shown. Note that most of the SNPs within the *GSTT2B* deletion are located in the inverted repeat (gray lines with arrowheads). Bar diagrams indicate % of individuals homozygous for each deletion who was genotyped as null for each SNP. B. Linkage disequilibrium (LD) of deletion polymorphisms with SNPs. LD (*r^2^*) plots are shown for the *GSTT2B* (red) and *GSTT1* deletion polymorphisms (blue) in European (CEU), Japanese and Chinese (JPT+CHB), and Yoruba (YRI) populations.

Despite its high frequency, the *GSTT2B* deletion polymorphism was not detectable by systematic methods using the HapMap SNP genotypes [1],[5]; which raises the question of SNP genotypes within the DNA-IR. HapMap SNP density is lower than average within this locus: 37 SNPs within 124 kb in European (CEU) samples (1 SNP/3.3 kb) (Figure 1A). In order to obtain SNP genotypes within the *GSTT2B* deletion polymorphism, we determined the genotype of *GSTT2B* deletion in the HapMap samples (Table 2) (Table S1). The *GSTT1* deletion genotype was determined previously for the HapMap samples [5]. The frequency of the *GSTT2B* deletion allele was very high in CEU (0.63), which is consistent with that of our Caucasian samples. The deletion polymorphism of *GSTT2B* spans 7 SNPs, 6 of which are located within the duplicated segment, while the *GSTT1* deletion, which can be correctly identified by SNP-based methods, contains 11 SNPs (Figure 5A) (Table S2). For each sample, SNP genotypes were obtained from the HapMap website. We expected a null genotype (N/N) in case of homozygous deletion. In fact, this was the case for the *GSTT1* deletion, in which two SNPs (rs2266633 and re5760170) were assigned with null genotypes in more than 50% of the 15 CEU individuals with homozygous deletion. Fifteen individuals (100%) were genotyped as null for rs2266633, indicating excellent “SNP tagging” of the *GSTT1* homozygous deletion. In contrast, none of the SNPs correctly genotyped the 39 individuals who are homozygous for *GSTT2B* deletion. One SNP (rs9608219) that was located outside of the duplicated region was called as null in 5 individuals (11.6%), while one individual was genotyped as null for rs2330649. None of the other SNPs were genotyped as null. Therefore, the *GSTT2* deletion polymorphism status could not be genotyped correctly by the assay used for the HapMap SNP genotypes, which strongly suggests a difficulty of correctly genotyping deletions located within a recently duplicated region using SNP-based approach [19],[20].

**Table 2:** GSTT2B and GSTT1 deletion polymorphisms in HapMap samples.

Genotypes					
Population	GSTT2B_Del/GSTT2B_Del	GSTT2B/GSTT2B	GSTT2B_Del/GSTT2B	samples (n)	HWE(p-value)
CEU	25	9	26	60	0.587
JCP	26	26	37	89	0.1368
YRI	11	14	35	60	0.299

Population	GSTT1_Del/GSTT1_Del	GSTT1/GSTT1	GSTT1_Del/GSTT1	samples (n)	HWE(p-value)
CEU	9	29	22	60	1
JCP	37	11	41	89	1
YRI	20	12	28	60	0.7926

Haplotypes								
Population	Haplotype	Freq	S.E	D	D′	Corr	Chisq	P-value
CEU (n = 60)				0.1875639	0.8408842	0.79739	76.2988	<2.2204e-16
	GSTT2B_DEL-GSTT1	0.558281	0.00080					
	GSTT2B_DEL-GSTT1_DEL	0.075052	0.00080					
	GSTT2B-GSTT1	0.050052	0.00080					
	GSTT2B-GSTT1_DEL	0.316614	0.00080					
JCP (n = 89)				0.1073	0.6061	0.4486	35.8186	2.17E-09
	GSTT2B_DEL-GSTT1	0.2813	0.01228					
	GSTT2B_DEL-GSTT1_DEL	0.2187	0.01228					
	GSTT2B-GSTT1	0.0726	0.01228					
	GSTT2B-GSTT1_DEL	0.4274	0.01228					
YRI (n = 60)				0.0235	0.1033	0.0949	1.0816	0.2983483
	GSTT2B_DEL-GSTT1_DEL	0.2564	0.01770					
	GSTT2B-GSTT1	0.2186	0.01770					
	GSTT2B-GSTT1	0.2147	0.01770					
	GSTT2B-GSTT1_DEL	0.3103	0.01770					

Freq, allele frequency; S.E., standard error; D, raw difference in frequency between observed number and expected number.

D′, scaled D spanning the range [−1,1]; Corr, Correlation Coefficient; chisq, Chisquare statistics for linkage equilibrium; p-value, Chi-square p-value for marker independence.

### Associations between deletion polymorphisms and SNPs differ among ancestries

The *GSTT2B* deletion polymorphism was also very common in both the Japanese/Chinese populations (JCP) and the Yoruba population (YRI), with an allele frequency of 0.50 and 0.47, respectively (Table 2). Since individuals' genotypes for *GSTT1* were available, we further addressed the association between *GSTT2B* and *GSTT1* deletion polymorphisms in HapMap populations. Consistent with the results from our Caucasian samples, LD between the two deletion polymorphisms was strong in CEU (*D′* = 0.841), with a significant overrepresentation of alleles with the single deletion (*p* = 2.2×10^−16^) (Table 2). In contrast, LD was less evident in JCP (*D′* = 0.60). Association of the two deletions appears to be random in YRI (*D′* = 0.10). In fact, data from SNP genotypes from HapMap samples in the surrounding region support our observations. There is a large haplo-block including two deletions in CEU (Figure S2). Phased haploblock analyses show that haplotypes in CEU are less diverse than in YRI (Figure S3).

In order to determine whether the *GSTT2B* deletion can be tagged by neighboring SNPs, we also assessed LD between the deletion polymorphisms and surrounding SNPs (Figure 5B) (Tables S3, S4, S5, S6, S7, and S8). HapMap SNP genotypes 500 kb to either side of deletions were obtained, and *r^2^* between deletion polymorphisms and SNPs was calculated. LD between the *GSTT2B* deletion polymorphism and SNPs were observed, and SNPs with *r^2^*>0.7 were identified up to 35 kb of the centromeric side and 11 kb on the telomeric side of the deletion in all three populations. There were several SNPs showing strong LD (*r^2^*>0.8) in JCP. Considering the fact that identifying SNPs showing complete LD (*r^2^* = 1.0) with nearby CNVs is very difficult in complex, repeat-rich regions [6],[47],[48], we may conclude that the *GSTT2B* deletion allele is tagged by nearby SNPs and is derived from a unique ancestral allele.

In contrast, LD between SNPs and the *GSTT1* deletion polymorphism showed a population-specific pattern. The deleted region including *GSTT1* is flanked by a pair of 466-bp direct repeat (Figure 2A). The 51-kb region between direct repeat is deleted in the deletion allele of *GSTT1* with only one 466-bp repeat remaining in the allele, which strongly suggests non-allelic homologous recombination (NAHR) as an underlying mechanism. SNPs with *r^2^*>0.7 were identified up to 100 kb on the centromeric side in CEU, consistent with the previous analysis [5]. In contrast, SNPs with *r^2^*>0.7 were less frequent and were only found within 10 kb on either side of the *GSTT1* deletion in JCP. There were no SNPs with *r^2^*>0.7 in YRI. Therefore, the *GSTT1* deletion would be found recurrently in humans, and extended LD between SNPs and *GSTT1* deletion polymorphism in CEU may be the result of selection forces for the haplotype harboring *GSTT1* deletion.

### Linkage disequilibrium between deletion polymorphisms in the human genome

We have observed CEU-specific LD between *GSTT2B* and *GSTT1* deletion polymorphisms. It is currently unknown whether closely located deletion polymorphisms are often in LD. Answering this question is very difficult, because, although a number of CNVs have been identified for the HapMap samples, the breakpoints as well as the copy-numbers for each CNV have not been well defined. Each CNV region tends to cover a large genomic region that may include more than one CNV. This is the case for the deletion polymorphisms for *GSTT2B* and *GSTT1*, in which a large single CNV region (cnp1364) covers both deletion polymorphisms [6].

Recently, very high-density microarray has begun to provide the locations of CNVs with higher resolution. McCarroll *et al.*, have developed an extremely high-density oligonucleotide microarray (Affymetrix SNP 6.0) and has captured CNVs in the HapMap samples with improved resolution [48]. Indeed, this approach captured *GSTT2B* (cnp id 2559) and *GSTT1* (2560) deletion polymorphisms as independent ones. Although the estimated size of the cnp 2559 is larger (67.1 kb, chromosome 22: 22,613,016–22,670,785) than the size from our direct sequencing of breakpoints, a genotype result for each individual is highly (100%) consistent with the results from PCR assay. Therefore, the data provided by McCarroll et al., would be valid for performing a genome-wide LD analysis.

In order to determine linkage between CNVs, we first selected the CNVs using the following criteria: 1) we focused on the diallelic deletion polymorphisms that are denoted as 0, 1 and 2 in the publication, which leave 361 polymorphisms; 2) we focused on deletion polymorphisms on autosomes and excluded 16 CNVs on sex chromosomes; and 3) we determined the linkage between CNVs that were on the same chromosomes. There were 1857 pairs (combinations) for CEU, 1734 for JPT+CHB and 2592 for YRI for linkage analysis, because some of the CNVs were only seen in one or two populations.

**Figure 6:**
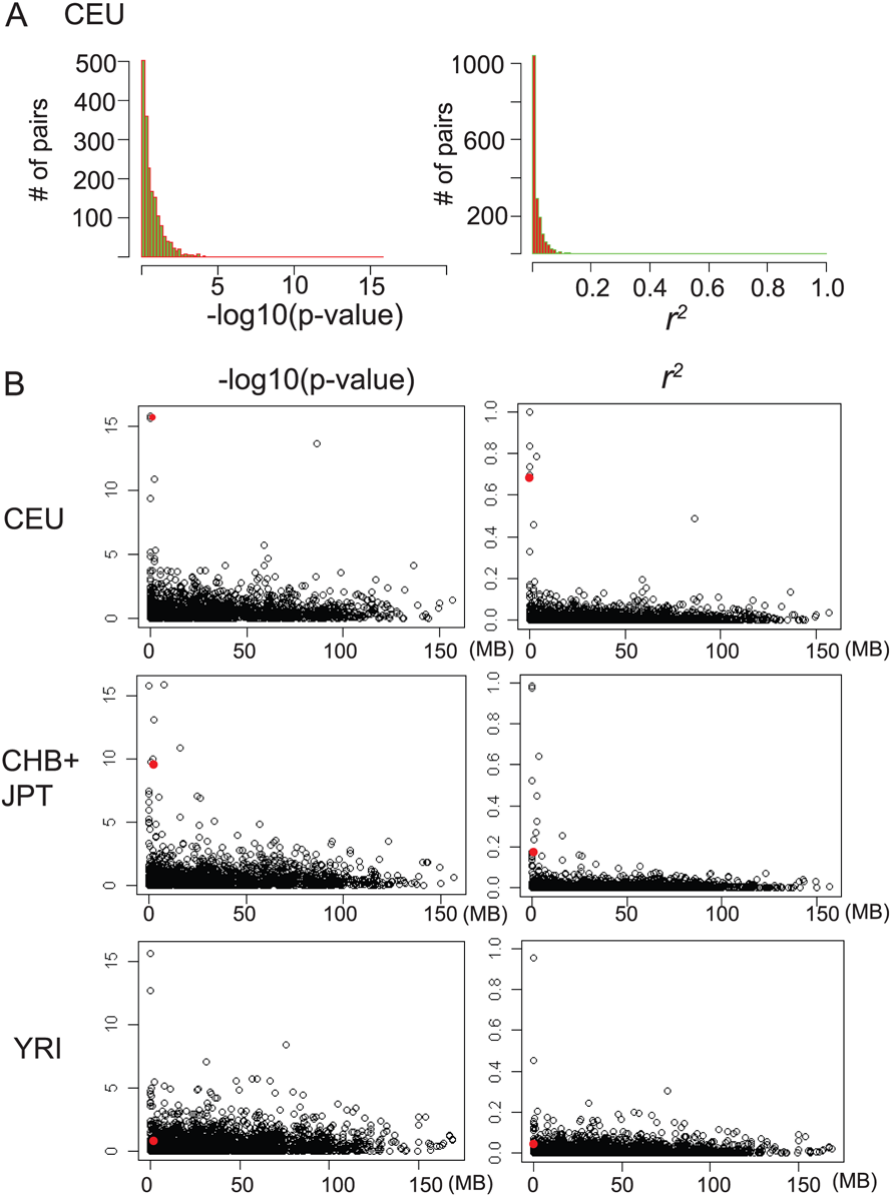
Pairwise linkage disequilibrium between deletion polymorphisms in the genome. A. The majority of deletion polymorphisms are not associated strongly with each other. Pairwise LD was shown for CEU population. The number of pairs (y-axis) were plotted against −log_10_(p-value) (top) and *r^2^* (bottom) (x-axis). B. Strong LD is uncommon between closely located deletion polymorphisms. For CEU (top), CHB+JPT (middle) and YRI (bottom), −log_10_(p-value) (left) and *r^2^* (right) (y-axis) are plotted against the distance between each pair of deletion polymorphisms. For each plot, the figures for the *GSTT2B-GSTT1* pair are indicated by a red circle.

First, we determined the number of deletion polymorphism pairs as a function of *r^2^* and significance value (−log_10_p-value) (Figure 6A, only for CEU). For both *r^2^* and significance value, the number of pairs showed power-law distributions and the vast majority of pairs had very low *r^2^* and −log_10_(p-value). This indicates that only a small number of deletion polymorphisms are in LD. However, consistent with the result from our PCR-genotyping, *GSTT2B-GSTT1*in CEU was in a strong LD (*r^2^* = 0.699, −log_10_(p-value)>15 ) (Figure 6B, marked with red circles) (Tables S9). Next, in order to determine whether strong LD was common for closely located CNVs, we determined the *r^2^* and significance value as functions of physical distance (Figure 6B). In fact, there were several, closely located deletion polymorphism pairs with relatively high *r^2^* (Tables S9, S10, and S11). These pairs were seen mostly in CEU and CHB+JPT, but not in YRI. Overall, there was very weak association for most of the pairs, even for the ones that are closely located. Therefore, the analysis using the currently available list of deletion polymorphisms indicates that the strong LD between *GSTT2B* and *GSTT1* in CEU seems unique and may imply the presence of selection forces in this locus.

## Discussion

Deletion alleles of *GST* genes have been known for more than a decade, long before we realized the global distribution and significant impact of CNVs on genetic variation in humans. Without knowing the major role of CNVs in genetic variation, deletion polymorphisms of *GST* genes might well have been accepted as common polymorphisms in humans but a rare event in the human genome. Knowing now both the prevalence of CNVs and the location of *GST* genes in extensive SDs, we may need to consider a more detailed genotyping of *GST* genes for disease association studies. Our approach using Restriction Fragment Length Polymorphisms (RFLP) illustrated an overall genetic diversity within the *GSTT2*-*GSTT1* locus. Two major common variants were evident in our analysis: a *GSTT2B*-deletion allele and a *GSTT1*-deletion allele. The *GSTT2B* deletion extended for 37 kb and caused a nearly silenced expression of the remaining *GSTT2*. Therefore, a null allele likely exists for both of the theta class of *GST* genes in humans.

Our study revealed the high frequency of the *GSTT2B* deletion alleles in all three HapMap populations, particularly in the CEU population. This is in contrast to the neighboring *GSTT1* deletion that is the least common in Caucasians [5]. Therefore, if there are any confounding effects of the *GSTT2B* deletion in the *GSTT1* disease association studies, it would affect associations in Caucasians more than in other populations. Association studies between lung cancer susceptibility and *GSTT1* deletion may illustrate this issue. Cigarette smoke is the main environmental risk factor for lung cancer. Cigarette smoke contains free radicals and induces oxidative damage to cellular lipids and DNA [49]. The theta class of GST exhibits glutathion peroxidase activity that protects cells from oxidative damage [50]. Recent meta-analyses show a marginal, but positive correlation between *GSTT1* deletion and lung cancer for Asians, but not for Caucasians [36],[38],[51]. We could speculate a possible reason for this observation: high frequency of the *GSTT1* homozygous deletion (40–60%) and lower *GSTT2B* deletion in Asians may have lead to a more accurate, positive association, whereas significant associations were difficult to find in Caucasians due to low frequency of (10–20%) the *GSTT1* deletion and high frequency of the *GSTT2B* deletion. Therefore, evaluating *GSTT2B* deletion polymorphism may be necessary in order to accurately assess associations between theta class of GST and human diseases in the future.

One of the unique features for the *GSTT2B* and *GSTT1* deletion polymorphisms is strong LD in the CEU population. Only a small number of deletion polymorphisms are in LD among the currently defined deletion polymorphisms. However, this conclusion is preliminary, given the fact that the dataset we used has a limited coverage on CNVs, in particular on smaller (<5 kb) ones [48]. DNA sequence level information on CNVs [13] for a large number of individuals is necessary in order to provide an improved list of CNV pairs with strong LD. One can do this for particular pairs by developing a PCR assay for each CNV based on the sequence of breakpoints and determine if there is any strong LD between CNVs. A CNV-based assessment of LD may be useful to complement the SNP-based approach, particularly for complex loci. Because the density of reliable SNPs may be limited in complex loci, a SNP-based approach may not have enough power for reliably assessing LD.

Among other pairs of deletion polymorphisms, LD was very strong in pairs of deletion polymorphisms that are located in peri- centromeric regions (Tables S9, S10, and S11). Low recombination rate within peri-centromeric region [52] would contribute to the strong LD. For example, both CNV 796 and 797 are located within the 80 kb peri-centromeric region of the short arm of chromosome 5. The frequencies of deletion alleles are very high in all three populations (796 – 0.45 in CEU, 0.41 in JCP and 0.25 in YRI; 797 – 0.45 in CEU, 0.41 in JCP and 0.25 in YRI). However, in contrast to the *GSTT2B-GSTT1* deletion polymorphism, LD is extremely strong in all three populations (*r^2^*; 0.999 in CEU, 0.985 in JCP and 0.955 in YRI). Deletions would occur very early in the history of humans and have been kept in the different alleles due to the lack of recombination. This emphasizes the uniqueness of deletions, and may further support the history of selection in shaping CEU-specific LD between *GSTT2B-GSTT1* deletions.

A distinct pattern of LD with nearby SNPs was seen for each deletion. The *GSTT2B* deletion appears to be tagged by nearby SNPs in all three populations. In contrast, CEU-specific, extended LD with SNPs was seen for the *GSTT1* deletion. The *GSTT2* deletion polymorphism most likely occurred after human-chimpanzee divergence and the deletion allele might have been propagating within the human linage. In contrast, linkage equilibrium between the *GSTT1* deletion and nearby SNPs in YRI strongly suggests that the deletion including *GSTT1* have occurred recurrently in humans, possibly by NAHR between 466-bp direct repeat. In CEU, the *GSTT1* deletion is almost exclusively seen in the allele that retains *GSTT2B*. Therefore, a potential scenario could be that the *GSTT1* deletion occurred in the *GSTT2B* non-deletion allele and has been selected for within CEU. The *GSTT1* deletion could also be selected in JCP population. However, because *GSTT1* deletion might have occurred recurrently in the two major alleles, the *GSTT2B* deletion allele and non-deletion allele in JCP, LD with nearby SNPs would not be as evident as in CEU.

We initiated this study on the assumption that the instability of large DNA-IRs may be a predisposing factor for CNVs. For example, a duplicated transgene in a 16 kb perfect palindrome (DNA-IR) in mice was transmitted to the progeny with very high frequency of DNA rearrangements (>15%) [53]. Typically, DNA rearrangements occur as a deletion of a tip and part of a DNA-IR. It was shown that nuclease processing of either a tip of hairpin structure on the lagging-strand DNA during replication resulted in two-ended DNA breaks [54]. Subsequent end joining may complete the deletion process. The *GSTT2B* deletion includes a part of spacer and one entire repeat, which is consistent with the proposed mechanism. However, from our results, we do not know whether rearrangements occur very frequently in this particular DNA inverted repeat. The high frequency of the *GSTT2B* deletion most likely comes from a unique allele propagating in humans, because these alleles likely share an identical breakpoint. This inverted repeat may not be as unstable as perfect DNA palindromes due to the presence of a 2.1-kb non-palindromic spacer and the sequence divergence (2.1%) between repeats. However, it still is of note that there is one individual (1/44) who has an atypical deletion (Figure 1). Therefore, overall genotypes of the locus could be more diverse than is described here.

We found severely reduced expression of the *GSTT2* gene in cell lines with homozygous *GSTT2B* deletion, suggesting an influence on neighboring gene expression [55]. Coggan et al., have shown previously that the *GSTT2B* gene has a mutation at the exon 2/intron 2 splice site that causes a premature termination at codon 196 in 28% of the Australian population. This allele was considered as a nonfunctional pseudogene (*GSTT2P*) [56]. We have also observed the same mutation in a subset of our samples from Caucasian (9/19) and African (2/10) individuals (data not shown). However, regardless of the functional status (*GSTT2B* or *GSTT2P*), the presence of the second *GSTT2* copy and its surrounding region have potential functional influence over *GSTT2* expression. Position effect may explain the reduced expression. A single functional enhancer for the pair of *GSTT2(B)* genes could potentially reside in the deleted region. The deletion would take out the single major positive control element and leave *GSTT2* inactive. Alternatively, DNA-IRs itself may have a positive synergistic effect on gene expression. Gene amplification of a drug resistance gene is very often initiated by inverted duplication [57]. Inverted duplications occur to counteract specific inhibitors by increasing copy number and gene expression. Although the unstable nature of DNA-IRs has been widely recognized, a number of large stably maintained DNA inverted repeats in the human genome [39] may also suggest an advantage of DNA-IRs in biological processes, such as gene expression and DNA replication. It is important to note that, in fibroblasts, *GSTT2* is reported as a differentially expressed gene between humans and chimpanzees [58], with a much higher level of expression in the chimpanzee.

In contrast to humans, chimpanzees strictly retained both *GSTT1* and *GSTT2B* genes in the samples tested here. Consistent with our finding, a previous study has not identified CNVs for these two genes in chimpanzees [59], although the study was done using BAC-clone based array-CGH analysis with limited resolution (1 MB). Our results provide specific genes involved in a lineage-specific CNV, which allows us to discuss history and function of the CNV. The conserved local genomic feature (DNA-IR) between two species, but frequent CNVs only in humans suggests the involvement of recent selective pressure. The theta-class is considered to be the most ancestral class of cytosolic GSTs, and other classes, such as mu (*GSTM*), alpha (*GSTA*) and pi (*GSTP*), originated from the theta class by gene duplication [33]. Importantly, unlike alpha and mu classes that have four and five paralogues respectively, there are only two paralogues for the theta-class, *GSTT1* and *GSTT2*. Why then are we losing (functionally) one of the most conserved classes of cellular detoxification genes? The answer may be that the theta class is dispensable due to the overlapping functions with other classes. However, there are several structural features that indicate a distinct function of the theta-class [60],[61]. First, amino acid identity between the theta-class and other classes is very low, less than 15% in mammals. Second, the highly conserved Tyr residue, a critical residue for glutathione (GSH) binding in other classes, is replaced by Ser. Third, the C-terminal extension in the theta-class proteins completely buries the substrate-binding pocket and occludes most of the GSH-binding site. Accordingly, the mammalian theta class lacks the ability to bind to glutathione affinity matrices, and lacks the activity with a model substrate of GSTs, 1-chloro-2,4-dinitrobenzene (CNDB). The least accessible substrate-binding site may indicate a much narrower range of substrates, which is in contrast to other classes that possess more open, accessible substrate binding sites for a wide range of substrates. Therefore, the compromised ability to detoxify theta-class specific substrates in humans may be related to the difference in phenotypes between two species [62].

In summary, we have characterized a high frequency deletion polymorphism of *GSTT2B* in a complex region of the genome. We provided a molecular approach in order to directly genotype the *GSTT2B* deletion, which may be useful for future disease association studies. These results confirm the unusual genetic and molecular features in the regions of segmental duplications, and the necessity of a labor-intensive approach for full understanding of the biology and disease phenotypes associated with CNVs.

## Materials and Methods

### Samples

Peripheral-blood cells, EBV-transformed lymphoblast cell lines, and DNA samples were collected from healthy donors [63]. Informed consent was obtained from all subjects in accordance with procedures and protocols approved by Human Subjects Protection Committee. HapMap DNA samples were obtained from the Coriell Institute (http://www.coriell.org/). Sample ID and *GSTT2B* genotype are listed in the Table S1.

Colorectal cancer cell lines HCT116, Lovo, HCT15, and HT29 were obtained from the ATCC. Ovarian cancer cell lines 2008 and 2008 (C13) were gift from Dr. Toshiyasu Taniguchi (Fred Hutchinson Cancer Research Center).

Human primary fibroblasts (AG16409, AG10803, AG09319, AG09309 and AG09429), Chimpanzee primary fibroblasts (AG06939, S003642, S003649, S006007, S007603) and lymphoblastoid cell lines (AG18354, AG18355, AG18356, AG18357, AG18358, AG18359, AG16618) were obtained from the Coriell Institute.

### DNA analysis

High molecular weight genomic DNA was extracted by QIAamp DNA Blood Midi kit (QIAGEN). Southern blotting was carried out as described previously [64]. Two µg of high-molecular-weight human genomic DNA were digested with a restriction enzyme, separated in 0.8% agarose gels. The gel was transferred to a positively charged nylon membrane (Amersham Biosciences) for 3 h at 75–80 mmHg pressure using the PosiBlot 30–30 pressure blotter and pressure control station (Stratagene). The DNA was UV-crosslinked to the nylon membrane using the Stratalinker 1800 UV crosslinker (Stratagene). To make a probe for Southern-blot analysis, we amplified human genomic DNA using PCR primers IR28-26352F, 5′-CAAGAGGCTACACAGGCAGATGTC-3′, IR28-26980R 5′-GGGCAGAGGAACGGAAACA-3′, and cloned the fragment by TOPO TA Cloning Kit (Invitrogen).

In order to genotype the *GSTT2B* deletion, a three primer set was designed for PCR: GSTT2B-6858, 5′-CACTCAACACAGTAGCCTCATCGTG-3′, *GSTT2B*-6857, 5′ TGCCTCCCCTGCCTTATTTC 3′, and *GSTT2B*-2B, 5′-CCTTCTGAAATGGAGCCTTTG-3′. The reaction was performed in a duplex-PCR with a final volume of 50 µl with 1.0 U Taq polymerase (GoTaq, Promega), 1.5 mM MgCl_2_, 200 µM dNTPs, 10 pmol of each primer, and 50 ng of genomic DNA. The thermal cycling conditions used for amplification consisted of an initial denaturation step at 95°C for 2 min, followed by 30 cycles of denaturation at 95°C for 30 s, annealing at 60°C for 30 s, and extension at 72°C for 45 s. Duplex PCR analysis for *GSTT1* was performed using the four primers as previously reported [45]. The reaction was performed in the final volume of 25 µl with 0.4 U of Faststart Taq polymerase, GC rich solution (Roche, USA), 2 mM MgCl_2_, 800 µM dNTPs, 10 pmol of each oligonucleotide primer, and 50 ng of genomic DNA. Thermal-cycling conditions consisted of an initial denaturation step at 95°C for 7 min, followed by 30 cycles of denaturation at 95°C for 30 s, annealing at 60°C for 30 s, and extension at 72°C for 60 s, and final extension at 72°C for 7 min.

Automated sequencing was performed directly both on the gel-purified PCR products and the PCR product cloned into TOPO TA cloning kit (Invitrogene, USA).

### mRNA analysis

Total RNA was extracted from cells using the RNeasy Kit (Qiagen). Ten µg of total RNA was loaded onto a 0.9% agarose-formaldehyde gel and separated for 60 min at 100 V. RNA quality was assessed by the integrity of 28S and 18S. The gel was transferred to a positively charged nylon membrane (Amersham Biosciences) for 3 h at 75–80 mmHg pressure using the PosiBlot 30–30 pressure blotter and pressure control station (Stratagene). The RNA was UV-crosslinked to the nylon membrane using the Stratalinker 1800 UV crosslinker (Stratagene). Each membrane was probed for both *GSTT2* and *β-actin*. Both probes were PCR amplified and cleaned using the Gel Extraction Kit (Qiagen). Primers for the GSTT2 cDNA are GSTT2 cDNA 31F – 5′-AGAGCTGTTTCTTGACCTGGTGTC-3′, GSTT2 cDNA 938R – 5′-GGTTATGTATGCTGCACCTGAGG-3′. Each probe was labeled with [α-^32^P]dATP (3000 Ci/mmol; Perkin Elmer). Membranes were hybridized overnight at 65°C in modified Church Buffer (0.5 M sodium phosphate, pH 7.2, 7% SDS, 10 mM EDTA) and exposed to Kodak BioMax MS film (Kodak). After probing for *GSTT2*, membranes were stripped at 65°C for 2 h in 0.5% SDS and reprobed for *β-actin* to verify equal amounts of RNA in each lane.

1 to 2 µg of RNA was reverse transcribed using the Superscript First-Strand Synthesis kit (Invitrogen) according to the manufacturer's conditions. The real time PCR was carried out using a MiniOpticon Real Time PCR Detection System (Bio-Rad). The PCR reaction contained 50 ng/µl of cDNA, 10 pmol of each of the specific primer sets for *GSTT2* and *RPL32*, 6.25 µl of iQ SYBR Green Supermix (Bio-Rad) master mixture (2× mix containing 50 U/ml iTaq DNA polymerase, 6 mM MgCl_2_, SYBR Green I, dNTP mix, 20 nM fluorescein and stabilizers) in a final reaction volume of 13 µl. All reactions were performed in triplicate. Thermalcycling conditions for *GSTT2* consisted of an initial denaturation of 10 min at 95°C, 40 cycles of 15 s at 95°C denaturing and 1 min at 55°C annealing and a final extension step for 10 min at 72°C. Cumulative fluorescence was measured at the end of each of the 40 cycles. For *RPL32*, thermalcycling conditions consisted of an initial 2 min at 50°C and 10 min at 95°C, followed by 40 cycles of 15 s at 95°C denaturing and 1 min at 60°C annealing. Cumulative fluorescence was measured after each of the 40 cycles. Product specific amplification was confirmed by melting curve analysis. Primers used for quantification were as follows: *GSTT2*, forward, 5′-CGCTCAAGGATGGTGATTTC-3′ and reverse, 5′-AGGTACTCATGAACACGGGC-3′; *RPL32*, forward, 5′-GCCAGATCTTGATGCCCAAC-3′ and reverse, 5′-CGTGCACATGAGCTGCCTAC-3′. Relative quantification of *GSTT2* gene expression was determined by construction of a relative expression calibration curve using serial dilutions of a positive control.

### SNPs and LD analysis

The SNP genotypes used in this work were downloaded from HapMap Public Release #23a (2008-04-01). SNP genotypes were obtained for 500 kb regions to either side of deletions. *GSTT1* deletion genotypes for HapMap samples were obtained from the previous publication [5]. Haplotypes were determined using Phase 2.1 [46]. Hardy-Weinberg Equilibrium tests (HWE test), pairwise-r^2^ value, D and D′, Chi-square p-value for marker independence were computed using R (genetics package).

Association between CNVs was determined using the data by McCarroll *et al.* [48]. In this dataset, the locations of CNVs as well as genotypes of HapMap individuals were available. In this analysis, associations between deletion polymorphisms that are on the same autosomes were determined. For the 1857 pairs (combinations of deletion polymorphisms) for CEU, 1734 for JPT+CHB and 2592 for YRI, pairwise-*r^2^* value, D and D′, Chi-square p-value and Hardy-Weinberg Equilibrium tests (HWE test) were computed using R (genetics package). In order to determine the distance between two deletion polymorphisms, we used a formula, (|S1–S2|+|E1–E2|)/2, where S1 and S2 represent the start sites (hg18) of the CNVs and E1and E2 represent the end sites of the CNVs. Chi-square p-value and *r^2^* was plotted as a function of distance.

### Web resources

UCSC genome Browser, http://genome.ucsc.edu/

PipMaker and MultiPipMaker, http://pipmaker.bx.psu.edu/pipmaker/

The R Project for Statistical Computing, http://www.r-project.org/

PHASE: software for haplotype reconstruction, and recombination rate estimation from population data, http://stephenslab.uchicago.edu/software.html

## Supporting Information

Figure S1 Southern blotting analysis with a probe for three SfiI+NdeI fragments. Restriction map with the locations of the *GSTT2B*, *GSTT2* and *GSTT1* gene is shown. The probe (a small rectangle) hybridized to three fragments: left repeat of the DNA-IR (11.3 kb), right repeat of the DNA IR (9.7 kb) and the fragment near *GSTT1* (21 kb). Genomic DNA from 14 individuals (also shown in the Figure 1B, right panel) are shown. While a 9.7 kb fragments (corresponding to *GSTT2*) are retained in all individuals, a 11.3 kb fragment (corresponding to *GSTT2B*) are seen in 5 individuals.
(1.96 MB EPS)

Figure S2 Haploblocks for three populations (from HapMap website).
(4.74 MB EPS)

Figure S3 Phased Haplotype for three populations (from HapMap website).
(1.65 MB EPS)

Table S1 *GSTT2B* deletion genotypes (HapMap).
(0.04 MB XLS)

Table S2 Deletion (*GSTT2B* and *GSTT1*) and SNP genotypes (HapMap CEU).
(1.95 MB XLS)

Table S3 Linkage analysis between *GSTT2B* deletion and SNP (CEU).
(0.09 MB XLS)

Table S4 Linkage analysis between *GSTT1* deletion and SNP (CEU).
(0.41 MB XLS)

Table S5 Linkage analysis between *GSTT2B* deletion and SNP (JCP).
(0.16 MB XLS)

Table S6 Linkage analysis between *GSTT1* deletion and SNP (JCP).
(0.16 MB XLS)

Table S7 Linkage analysis between *GSTT2B* deletion and SNP (YRI).
(0.45 MB XLS)

Table S8 Linkage analysis between *GSTT1* deletion and SNP (YRI).
(0.17 MB XLS)

Table S9 Linkage analyses between deletion polymorphisms (CEU).
(0.18 MB XLS)

Table S10 Linkage analysis between deletion polymorphisms (JCP).
(0.16 MB XLS)

Table S11 Linkage analysis between deletion polymorphisms (YRI).
(0.23 MB XLS)
